# Bis[μ-4-(4-carb­oxy­phen­oxy)phthalato]bis­[triaqua­nickel(II)]

**DOI:** 10.1107/S1600536810049718

**Published:** 2010-12-11

**Authors:** Xue Cai

**Affiliations:** a Department of Chemistry, Mudanjiang Normal College, Mudanjiang 157012, Heilongjiang Province, People’s Republic of China

## Abstract

In the centrosymmetric binuclear title compound, [Ni_2_(C_15_H_8_O_7_)_2_(H_2_O)_6_], the Ni^II^ ion is in a distorted octa­hedral coordination geometry with O_6_ donors, three from three water mol­ecules, the others from three carboxylate groups of two ligands. Extensive O—H⋯O hydrogen bonding connects the mol­ecules into a three-dimensional supra­molecular structure.

## Related literature

For metal-organic coordination polymers, see: Evans *et al.* (1999 ▶); Li *et al.* (2008 ▶). For related structures, see: Wang *et al.* (2010 ▶); Hökelek *et al.* (2009 ▶).
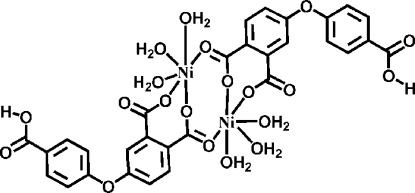


## Experimental

### 

#### Crystal data

[Ni_2_(C_15_H_8_O_7_)_2_(H_2_O)_6_]
*M* *_r_* = 825.90
Monoclinic, 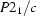

*a* = 14.4173 (9) Å
*b* = 9.5002 (6) Å
*c* = 11.2857 (7) Å
β = 92.632 (1)°
*V* = 1544.14 (17) Å^3^
*Z* = 2
Mo *K*α radiation
μ = 1.32 mm^−1^
*T* = 298 K
0.18 × 0.12 × 0.05 mm

#### Data collection

Bruker SMART APEX CCD area-detector diffractometer
Absorption correction: multi-scan (*SADABS*; Sheldrick, 2003) ▶ *T*_min_ = 0.798, *T*_max_ = 0.937
7457 measured reflections
2716 independent reflections
2245 reflections with *I* > 2σ(*I*)
*R*_int_ = 0.030

#### Refinement

*R*[*F* ^2^ > 2σ(*F* ^2^)] = 0.030
*wR*(*F* ^2^) = 0.069
*S* = 1.02
2716 reflections
263 parameters
10 restraints
H atoms treated by a mixture of independent and constrained refinement
Δρ_max_ = 0.28 e Å^−3^
Δρ_min_ = −0.31 e Å^−3^

### 

Data collection: *APEX2* (Bruker, 2004 ▶); cell refinement: *SAINT-Plus* (Bruker, 2001 ▶); data reduction: *SAINT-Plus*; program(s) used to solve structure: *SHELXS97* (Sheldrick, 2008 ▶); program(s) used to refine structure: *SHELXL97* (Sheldrick, 2008 ▶); molecular graphics: *XP* (Sheldrick, 1998 ▶); software used to prepare material for publication: *SHELXL97*.

## Supplementary Material

Crystal structure: contains datablocks global, I. DOI: 10.1107/S1600536810049718/ds2071sup1.cif

Structure factors: contains datablocks I. DOI: 10.1107/S1600536810049718/ds2071Isup2.hkl

Additional supplementary materials:  crystallographic information; 3D view; checkCIF report

## Figures and Tables

**Table 1:** Hydrogen-bond geometry (Å, °)

*D*—H⋯*A*	*D*—H	H⋯*A*	*D*⋯*A*	*D*—H⋯*A*
O6—H6*A*⋯O1^i^	0.85 (1)	1.73 (1)	2.579 (3)	176 (4)
O8—H8*A*⋯O10^ii^	0.85 (1)	2.41 (3)	2.967 (3)	124 (3)
O8—H8*B*⋯O2^iii^	0.86 (1)	2.07 (2)	2.841 (3)	150 (4)
O9—H9*B*⋯O3^iv^	0.84 (1)	2.14 (2)	2.889 (2)	149 (3)
O8—H8*A*⋯O7^v^	0.85 (1)	2.12 (2)	2.867 (3)	146 (3)
O10—H10*B*⋯O1^vi^	0.84 (1)	1.96 (1)	2.770 (3)	164 (3)

Symmetry codes: (i) 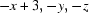; (ii) 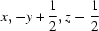; (iii) 
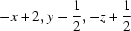
; (iv) 
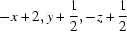
; (v) 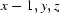; (vi) 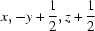.

**Fig. 1.**
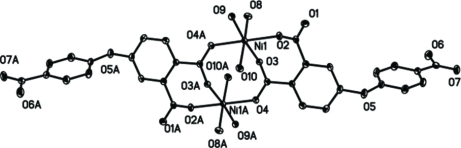
A view of (I) with the unique atom-labelling scheme. Displacement ellipsoids are drawn at the 30% probability level.

**Fig. 2.**
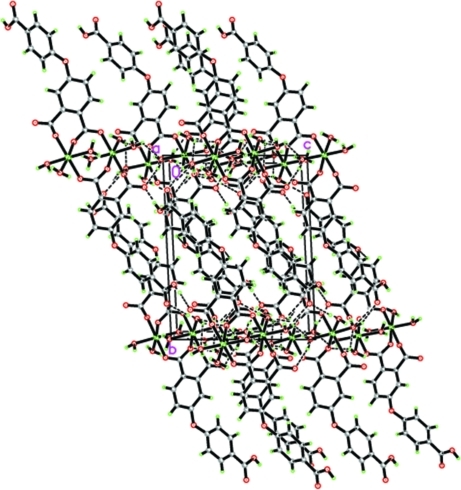
A packing diagram of (I) along a axis.

## supplementary crystallographic information

### Comment

In the field of supramolecular chemistry and crystal engineering, the design and assembly of metal-organic coordination polymers with appealing structures and properties have stimulated interests of chemists (Evans *et al.*, 1999). The hydrogen bonding interaction often leads to complicated spramolecular structure (Li *et al.*, 2008).

As shown in Fig.1, compound I is a new binuclear neutral complex with a shuttle molecular configuration. The two Ni(II) ions locate in the middle of this molecule. Ni(II) atom is coordinated in a octahedral coordination sphere The bond lengths of Ni—O are similar with the values in those complexes containing Ni—O sgment (Wang *et al.*, 2010). There are rich hydrogen bonding interaction O—H···O in this compound, giving a three-dimensional supramolecular structure.

### Experimental

H3L4 (0.0302 g, 0.1 mmol), Ni(OAc)_2_.4H_2_O (0.0498 g, 0.2 mmol), and H2O (15 ml) was sealed in 25 ml Teflon-lined stainless steel reactor and heated to 120 *^o^*C. Green block-shaped crystals suitable for X-ray diffraction analysis were separated by filtration with the yield of 37%

### Refinement

All H-atoms bound to carbon were refined using a riding model with distance C—H = 0.93 Å, *U*_iso_ = 1.2*U*_eq_ (C) for aromatic atoms. The distance of O—H of water molecule has been restrained using the '*DFIX*' command as 0.85Å with the deviation of 0.01.

### Crystal data

**Table:**

[Ni_2_(C_15_H_8_O_7_)_2_(H_2_O)_6_]	*F*(000) = 848
*M**_r_* = 825.90	*D*_x_ = 1.776 Mg m^−^^3^
Monoclinic, *P*2_1_/*c*	Mo *K*α radiation, λ = 0.71073 Å
Hall symbol: -P 2ybc	Cell parameters from 2290 reflections
*a* = 14.4173 (9) Å	θ = 2.6–25.3°
*b* = 9.5002 (6) Å	µ = 1.32 mm^−^^1^
*c* = 11.2857 (7) Å	*T* = 298 K
β = 92.632 (1)°	Sheet, green
*V* = 1544.14 (17) Å^3^	0.18 × 0.12 × 0.05 mm
*Z* = 2	

### Data collection

**Table:**

Bruker SMART APEX CCD area-detector diffractometer	2716 independent reflections
Radiation source: fine-focus sealed tube	2245 reflections with *I* > 2σ(*I*)
graphite	*R*_int_ = 0.030
Detector resolution: 0 pixels mm^-1^	θ_max_ = 25.0°, θ_min_ = 1.4°
ω scans	*h* = −16→17
Absorption correction: multi-scan (*SADABS*; Sheldrick, 2003)	*k* = −7→11
*T*_min_ = 0.798, *T*_max_ = 0.937	*l* = −13→13
7457 measured reflections	

### Refinement

**Table:**

Refinement on *F*^2^	Primary atom site location: structure-invariant direct methods
Least-squares matrix: full	Secondary atom site location: difference Fourier map
*R*[*F*^2^ > 2σ(*F*^2^)] = 0.030	Hydrogen site location: inferred from neighbouring sites
*wR*(*F*^2^) = 0.069	H atoms treated by a mixture of independent and constrained refinement
*S* = 1.02	*w* = 1/[σ^2^(*F*_o_^2^) + (0.0295*P*)^2^ + 0.7208*P*] where *P* = (*F*_o_^2^ + 2*F*_c_^2^)/3
2716 reflections	(Δ/σ)_max_ = 0.001
263 parameters	Δρ_max_ = 0.28 e Å^−^^3^
10 restraints	Δρ_min_ = −0.31 e Å^−^^3^

### Special details

**Table:**

Geometry. All e.s.d.'s (except the e.s.d. in the dihedral angle between two l.s. planes) are estimated using the full covariance matrix. The cell e.s.d.'s are taken into account individually in the estimation of e.s.d.'s in distances, angles and torsion angles; correlations between e.s.d.'s in cell parameters are only used when they are defined by crystal symmetry. An approximate (isotropic) treatment of cell e.s.d.'s is used for estimating e.s.d.'s involving l.s. planes.
Refinement. Refinement of *F*^2^ against ALL reflections. The weighted *R*-factor *wR* and goodness of fit *S* are based on *F*^2^, conventional *R*-factors *R* are based on *F*, with *F* set to zero for negative *F*^2^. The threshold expression of *F*^2^ > σ(*F*^2^) is used only for calculating *R*-factors(gt) *etc*. and is not relevant to the choice of reflections for refinement. *R*-factors based on *F*^2^ are statistically about twice as large as those based on *F*, and *R*- factors based on ALL data will be even larger.

### Fractional atomic coordinates and isotropic or equivalent isotropic displacement parameters (Å^2^)

**Table:**

	*x*	*y*	*z*	*U*_iso_*/*U*_eq_	
C1	1.13696 (16)	−0.0011 (3)	0.4707 (2)	0.0205 (6)	
C2	1.16031 (17)	0.1901 (3)	0.2544 (2)	0.0217 (6)	
C3	1.23890 (17)	0.1474 (3)	0.3408 (2)	0.0219 (6)	
C4	1.22697 (16)	0.0685 (3)	0.4442 (2)	0.0210 (6)	
C5	1.30275 (18)	0.0466 (3)	0.5226 (2)	0.0298 (7)	
H5	1.2949	−0.0042	0.5918	0.036*	
C6	1.38951 (18)	0.0989 (3)	0.4998 (2)	0.0302 (6)	
H6	1.4394	0.0847	0.5536	0.036*	
C7	1.40112 (17)	0.1721 (3)	0.3963 (2)	0.0263 (6)	
C8	1.32712 (17)	0.1980 (3)	0.3173 (2)	0.0252 (6)	
H8	1.3360	0.2490	0.2484	0.030*	
C9	1.62538 (18)	0.2565 (3)	0.2812 (2)	0.0313 (7)	
H9	1.6411	0.3291	0.3335	0.038*	
C10	1.54081 (17)	0.1884 (3)	0.2880 (2)	0.0237 (6)	
C11	1.51594 (18)	0.0824 (3)	0.2086 (2)	0.0313 (7)	
H11	1.4585	0.0383	0.2118	0.038*	
C12	1.57749 (18)	0.0433 (3)	0.1249 (2)	0.0306 (7)	
H12	1.5612	−0.0281	0.0715	0.037*	
C13	1.66329 (17)	0.1082 (3)	0.1185 (2)	0.0262 (6)	
C14	1.68622 (18)	0.2165 (3)	0.1967 (2)	0.0321 (7)	
H14	1.7429	0.2624	0.1921	0.039*	
C15	1.73084 (18)	0.0607 (3)	0.0322 (2)	0.0283 (6)	
O1	1.16730 (12)	0.1605 (2)	0.14741 (16)	0.0346 (5)	
O2	1.09321 (11)	0.25998 (18)	0.29324 (15)	0.0236 (4)	
O3	1.07414 (11)	−0.01111 (18)	0.38727 (14)	0.0215 (4)	
O4	1.12870 (11)	−0.04895 (19)	0.57313 (15)	0.0255 (4)	
O5	1.48851 (12)	0.2300 (2)	0.38073 (16)	0.0327 (5)	
O6	1.70082 (14)	−0.0415 (2)	−0.03746 (19)	0.0393 (5)	
O7	1.80790 (13)	0.1132 (2)	0.02540 (17)	0.0418 (6)	
O8	0.95069 (13)	0.0514 (2)	0.20299 (16)	0.0283 (4)	
O9	0.89424 (13)	0.3188 (2)	0.31920 (17)	0.0267 (4)	
O10	1.00957 (13)	0.2419 (2)	0.52416 (15)	0.0261 (4)	
Ni1	0.98127 (2)	0.15487 (3)	0.36037 (3)	0.01905 (11)	
H6A	1.7430 (19)	−0.079 (4)	−0.077 (3)	0.074 (13)*	
H8A	0.927 (2)	0.094 (3)	0.143 (2)	0.062 (11)*	
H8B	0.918 (2)	−0.023 (3)	0.211 (3)	0.102 (17)*	
H9B	0.916 (2)	0.385 (2)	0.279 (2)	0.066 (12)*	
H9A	0.872 (2)	0.352 (3)	0.3811 (16)	0.048 (10)*	
H10B	1.0610 (11)	0.275 (3)	0.548 (2)	0.035 (9)*	
H10A	0.9937 (19)	0.176 (3)	0.569 (2)	0.062 (12)*	

### Atomic displacement parameters (Å^2^)

**Table:**

	*U*^11^	*U*^22^	*U*^33^	*U*^12^	*U*^13^	*U*^23^
C1	0.0208 (13)	0.0174 (13)	0.0238 (14)	0.0048 (11)	0.0051 (11)	0.0013 (11)
C2	0.0208 (13)	0.0191 (14)	0.0257 (14)	−0.0032 (11)	0.0061 (11)	0.0045 (11)
C3	0.0213 (13)	0.0217 (14)	0.0230 (13)	0.0026 (11)	0.0033 (10)	−0.0034 (11)
C4	0.0197 (13)	0.0235 (14)	0.0201 (13)	0.0017 (11)	0.0041 (10)	0.0005 (11)
C5	0.0267 (14)	0.0371 (17)	0.0256 (15)	0.0034 (13)	0.0029 (11)	0.0068 (13)
C6	0.0180 (13)	0.0408 (17)	0.0317 (15)	0.0032 (13)	−0.0004 (11)	0.0024 (13)
C7	0.0169 (13)	0.0305 (16)	0.0318 (15)	−0.0028 (12)	0.0061 (11)	−0.0077 (13)
C8	0.0238 (14)	0.0290 (15)	0.0235 (14)	−0.0010 (12)	0.0086 (11)	0.0013 (12)
C9	0.0257 (15)	0.0352 (17)	0.0333 (15)	−0.0080 (13)	0.0024 (12)	−0.0097 (13)
C10	0.0159 (12)	0.0271 (15)	0.0286 (14)	0.0013 (11)	0.0049 (11)	0.0010 (12)
C11	0.0187 (14)	0.0328 (16)	0.0426 (17)	−0.0077 (13)	0.0060 (12)	−0.0086 (14)
C12	0.0265 (15)	0.0326 (16)	0.0330 (15)	−0.0030 (13)	0.0038 (12)	−0.0081 (13)
C13	0.0191 (13)	0.0329 (16)	0.0266 (14)	0.0009 (12)	0.0027 (11)	0.0024 (12)
C14	0.0195 (14)	0.0415 (17)	0.0358 (16)	−0.0075 (13)	0.0061 (12)	−0.0023 (14)
C15	0.0255 (15)	0.0367 (17)	0.0228 (14)	0.0047 (13)	0.0010 (11)	0.0052 (13)
O1	0.0294 (10)	0.0526 (13)	0.0222 (10)	0.0137 (10)	0.0042 (8)	0.0001 (10)
O2	0.0211 (9)	0.0208 (10)	0.0296 (10)	0.0030 (8)	0.0094 (8)	0.0028 (8)
O3	0.0207 (9)	0.0230 (10)	0.0208 (9)	0.0008 (8)	−0.0002 (7)	0.0022 (8)
O4	0.0226 (9)	0.0323 (11)	0.0218 (10)	−0.0029 (8)	0.0039 (7)	0.0063 (8)
O5	0.0202 (9)	0.0427 (12)	0.0359 (11)	−0.0064 (9)	0.0083 (8)	−0.0118 (9)
O6	0.0297 (11)	0.0466 (14)	0.0424 (13)	0.0023 (10)	0.0097 (10)	−0.0129 (11)
O7	0.0266 (11)	0.0649 (16)	0.0349 (12)	−0.0102 (11)	0.0120 (9)	−0.0035 (11)
O8	0.0373 (11)	0.0276 (11)	0.0199 (10)	−0.0020 (10)	−0.0003 (8)	0.0014 (9)
O9	0.0263 (10)	0.0260 (11)	0.0284 (11)	0.0038 (8)	0.0077 (9)	0.0054 (9)
O10	0.0287 (11)	0.0273 (11)	0.0223 (10)	−0.0050 (9)	0.0018 (8)	−0.0025 (9)
Ni1	0.01854 (18)	0.02068 (18)	0.01820 (18)	0.00061 (15)	0.00355 (12)	0.00130 (14)

### Geometric parameters (Å, °)

**Table:**

C1—O4	1.253 (3)	C10—O5	1.376 (3)
C1—O3	1.279 (3)	C10—C11	1.384 (4)
C1—C4	1.499 (3)	C11—C12	1.377 (4)
C2—O1	1.249 (3)	C11—H11	0.9300
C2—O2	1.268 (3)	C12—C13	1.387 (4)
C2—C3	1.516 (3)	C12—H12	0.9300
C3—C8	1.396 (3)	C13—C14	1.385 (4)
C3—C4	1.405 (3)	C13—C15	1.479 (4)
C4—C5	1.389 (3)	C14—H14	0.9300
C5—C6	1.381 (4)	C15—O7	1.223 (3)
C5—H5	0.9300	C15—O6	1.310 (3)
C6—C7	1.376 (4)	O2—Ni1	2.0708 (17)
C6—H6	0.9300	O3—Ni1	2.0817 (17)
C7—C8	1.381 (4)	O4—Ni1^i^	2.0491 (17)
C7—O5	1.393 (3)	O8—Ni1	2.0600 (18)
C8—H8	0.9300	O9—Ni1	2.0405 (19)
C9—C14	1.378 (4)	O10—Ni1	2.0490 (18)
C9—C10	1.386 (4)	Ni1—O4^i^	2.0491 (17)
C9—H9	0.9300		
			
O4—C1—O3	123.9 (2)	C14—C13—C15	120.1 (2)
O4—C1—C4	117.6 (2)	C12—C13—C15	120.9 (3)
O3—C1—C4	118.5 (2)	C9—C14—C13	120.3 (2)
O1—C2—O2	123.3 (2)	C9—C14—H14	119.9
O1—C2—C3	118.1 (2)	C13—C14—H14	119.9
O2—C2—C3	118.5 (2)	O7—C15—O6	122.7 (3)
C8—C3—C4	119.3 (2)	O7—C15—C13	123.0 (3)
C8—C3—C2	116.5 (2)	O6—C15—C13	114.3 (2)
C4—C3—C2	124.1 (2)	C2—O2—Ni1	119.60 (15)
C5—C4—C3	119.1 (2)	C1—O3—Ni1	118.68 (16)
C5—C4—C1	118.1 (2)	C1—O4—Ni1^i^	128.60 (16)
C3—C4—C1	122.8 (2)	C10—O5—C7	120.9 (2)
C6—C5—C4	121.4 (2)	C15—O6—H6A	113 (3)
C6—C5—H5	119.3	Ni1—O8—H8A	122 (2)
C4—C5—H5	119.3	Ni1—O8—H8B	114 (3)
C7—C6—C5	119.1 (2)	H8A—O8—H8B	105.6 (15)
C7—C6—H6	120.4	Ni1—O9—H9B	117 (2)
C5—C6—H6	120.4	Ni1—O9—H9A	110 (2)
C6—C7—C8	121.2 (2)	H9B—O9—H9A	109.2 (16)
C6—C7—O5	116.9 (2)	Ni1—O10—H10B	125.5 (19)
C8—C7—O5	121.7 (2)	Ni1—O10—H10A	102 (2)
C7—C8—C3	120.0 (2)	H10B—O10—H10A	109.7 (16)
C7—C8—H8	120.0	O9—Ni1—O10	89.55 (8)
C3—C8—H8	120.0	O9—Ni1—O4^i^	88.86 (7)
C14—C9—C10	120.0 (3)	O10—Ni1—O4^i^	89.61 (7)
C14—C9—H9	120.0	O9—Ni1—O8	93.61 (8)
C10—C9—H9	120.0	O10—Ni1—O8	175.12 (8)
O5—C10—C11	124.5 (2)	O4^i^—Ni1—O8	86.73 (8)
O5—C10—C9	115.0 (2)	O9—Ni1—O2	91.72 (7)
C11—C10—C9	120.4 (2)	O10—Ni1—O2	90.52 (7)
C12—C11—C10	119.0 (2)	O4^i^—Ni1—O2	179.40 (7)
C12—C11—H11	120.5	O8—Ni1—O2	93.11 (7)
C10—C11—H11	120.5	O9—Ni1—O3	174.95 (7)
C11—C12—C13	121.3 (3)	O10—Ni1—O3	94.25 (7)
C11—C12—H12	119.3	O4^i^—Ni1—O3	94.47 (7)
C13—C12—H12	119.3	O8—Ni1—O3	82.82 (7)
C14—C13—C12	119.0 (2)	O2—Ni1—O3	84.94 (7)

Symmetry codes: (i) −*x*+2, −*y*, −*z*+1.

### Hydrogen-bond geometry (Å, °)

**Table:**

*D*—H···*A*	*D*—H	H···*A*	*D*···*A*	*D*—H···*A*
O6—H6A···O1^ii^	0.85 (1)	1.73 (1)	2.579 (3)	176 (4)
O8—H8A···O10^iii^	0.85 (1)	2.41 (3)	2.967 (3)	124 (3)
O8—H8B···O2^iv^	0.86 (1)	2.07 (2)	2.841 (3)	150 (4)
O9—H9B···O3^v^	0.84 (1)	2.14 (2)	2.889 (2)	149 (3)
O8—H8A···O7^vi^	0.85 (1)	2.12 (2)	2.867 (3)	146 (3)
O10—H10B···O1^vii^	0.84 (1)	1.96 (1)	2.770 (3)	164 (3)

Symmetry codes: (ii) −*x*+3, −*y*, −*z*; (iii) *x*, −*y*+1/2, *z*−1/2; (iv) −*x*+2, *y*−1/2, −*z*+1/2; (v) −*x*+2, *y*+1/2, −*z*+1/2; (vi) *x*−1, *y*, *z*; (vii) *x*, −*y*+1/2, *z*+1/2.
